# Serum Magnesium and Vitamin D Levels as Indicators of Asthma Severity

**DOI:** 10.1155/2016/1643717

**Published:** 2016-10-12

**Authors:** Mohammed Nadeem Shaikh, Brahma Reddy Malapati, Ruchi Gokani, Bhavita Patel, Mitul Chatriwala

**Affiliations:** ^1^Department of Biochemistry, Parul Institute of Medical Science and Research (PIMSR), Limda, Waghodia, Gujarat 391760, India; ^2^Department of Biochemistry, C.U. Shah Medical Collage, Surendranagar, Gujarat, India; ^3^Department of Biochemistry, S.B.K.S MI & RC, Piparia, Vadodara, Gujarat 391760, India; ^4^Department of Biochemistry, Pramukhswami Medical College, Karamsad, Gujarat, India

## Abstract

*Background*. Serum magnesium levels affect the concentration of circulating vitamin D in blood and subsequently it affects the immunity; thus it plays significant role in the pathogenesis of asthma. Asthma, in adults, is less studied and hypomagnesemia along with vitamin D deficiency and insufficiency is common in asthmatic individuals, which causes frequent asthma attacks, respiratory infections, severe exacerbations, and poor response to bronchodilators.* Objective*. To detect the magnitude of vitamin D insufficiency and deficiency and serum magnesium levels among asthmatic patients and to correlate them with the severity of asthma.* Materials and Methods*. This is a cross-sectional case-control study which includes 60 patients of chronic stable asthma and 60 healthy controls. After taking clinical history and systemic examination, pulmonary function test was done. Serum levels of magnesium, 25-hydroxycholecalciferol [25(OH)D], and calcium were measured in all the subjects.* Results*. Significant correlation was found between vitamin D deficiency, hypomagnesemia, and asthma severity. Serum calcium levels were unaffected by that.* Conclusion*. Vitamin D and serum magnesium deficiency are highly prevalent in patients with asthma. Increased asthma severity, frequency of attacks, and exacerbation are associated with lower levels of one or both. Serum 25(OH)D and magnesium levels may serve as important markers of asthma severity.

## 1. Introduction

Bronchial asthma is a chronic condition characterised by recurrent bronchospasm resulting from reversible bronchial hyperresponsiveness in response to stimuli of a level or intensity which usually does not cause such narrowing in most individuals [1]. Globally bronchial asthma is one of the most common diseases which affects approximately ~334 million people. It has great social impact, with a prevalence of about 10–12% in adults and 15% in children. It can be found at any age [2].

Asthma is widely distributed all over the globe including tropical countries like India. But it has very variable prevalence among different populations. In India, its prevalence is about 2% [1, 2]. Because of its potential role in preventing respiratory infections, vitamin D deficiency is an important public health issue. The key function of vitamin D is to regulate calcium and phosphorus homeostasis and in turn vitamin D metabolism is regulated by factors that respond to plasma concentration of calcium, phosphate, and magnesium. Calcitriol (active form of vitamin D) is involved in insulin secretion, inhibition of interleukin production by T-lymphocytes and immunoglobulin by B-lymphocytes, differentiation of monocyte precursor cells, and modulation of cell proliferation. Cells of the immune system such as T-lymphocytes, activated B-lymphocytes, and dendritic cells express vitamin D receptors (VDRs) [3]. Dendritic cells also express 1,*α*-hydroxylase, which suggests that 25-hydroxycholecalciferol (Calcidiol) can be converted to its active form (Calcitriol) locally and so it participates in immune response. Moreover vitamin D is found to play a role in innate immunity against various microbial agents. It upregulates the synthesis of cathelicidin and immunoglobulins [4]. Probably this can be the possible reason behind the curing effects of sunlight on tuberculosis and other infections [5]. Thus, vitamin D is one of the important regulators of immunity. Its deficiency along with triggering stimuli may increase the risk of asthma, allergies, and exacerbation of diseases [6].

As per few earlier reports, magnesium (Mg) deficiency is associated with increased tracheobronchial hyperreactivity, pulmonary vascular drag, and ventricular arrhythmia [7, 8]. Treatment with *β*_2_-agonists can reduce magnesium levels in serum by intracellular shift or urinary loss [8]. Magnesium causes relaxation of bronchial smooth muscles and dilatation of airways, most probably by altering calcium ion movement. In contrary, hypomagnesemia may produce bronchoconstriction. It may disturb the neuromuscular mechanism to such an extent in certain individuals which may result in bronchial spasms [9]. Magnesium (Mg) as the second most plentiful intracellular cation and an important part of bone mineralization has a crucial function in synthesis and metabolism of vitamin D. Lower levels of serum magnesium are associated with hypovitaminosis D [10, 11]. Hypomagnesemia lowers the concentration of vitamin D in body which can worsen the clinical condition in asthma by tracheobronchial hyperreactivity and by increasing susceptibility to respiratory infections [4, 5, 7, 9, 10].

Hence, this study was carried out to detect the prevalence of hypomagnesemia with vitamin D insufficiency and deficiency among asthmatic patients of India. This study also aims to assess the relationship of serum Mg and vitamin D levels with severity of asthma.

## 2. Materials and Methods

### 2.1. Study Design

This is a cross-sectional case-control study carried out from May, 2014, to July, 2015. Study included 60 cases of chronic stable asthma as Group I and 60 healthy individuals (controls) matched with age, sex, and ethnic background as Group II. Patients with tuberculosis or any other associated respiratory/systemic disease, undergoing diuretic therapy, and of age <18 years were excluded from the study. Selection and classification of the patients were done according to the global initiative for asthma “GINA guidelines” [12]. A written informed consent was given by all the participants of the study prior to their inclusion. The Sumandeep ethics committee approved the study. Group I (asthmatic patients) included 60 patients, 38 (63.3%) males and 22 (36.7%) females. Their ages ranged from 19 to 71 years with a mean value of 40 ± 13 years. Group II (controls) included 60 healthy patients, 45 (75%) males and 15 (25%) females. Their ages ranged between 18 to 70 years with a mean value 42 ± 11 years. All the patients participating in the study were subjected to clinical history taking including medications. They underwent complete systemic examination. Blood samples were collected in plain vacuette (with clot activator) under aseptic conditions for measuring serum levels of magnesium, vitamin D (25(OH) D), and serum calcium. Finally pulmonary function test (PFT) by spirometry was done in each patient to obtain forced expiratory volume (FEV1), FVC, and PEF for determination of the severity (or stage) of asthma.

### 2.2. Analytical Methods

Serum 25(OH)D levels were measured by Chemiluminescent Immunoassay (CLIA) on Beckman Coulter, Access-2 instrument.

#### 2.2.1. Test Principle

Test kit is a two-step competitive binding immunoenzymatic assay. Sample is added to a reaction vessel with a DBP (vitamin D binding protein) releasing agent and paramagnetic particles coated anti-25(OH)D antibody from sheep. DBP releases 25(OH)D which binds to the fixed monoclonal anti-25(OH)D. Then analogue of 25(OH)D and alkaline phosphatase conjugate is added. It competes for binding to fixed monoclonal anti-25(OH)D. Molecules bound to paramagnetic particles gather in a magnetic field while unbound materials are washed away. Then, Lumi-Phos 530 (chemiluminescent substrate) is added and luminometer measures the light emitted by the reaction. The light production is inversely proportional to the concentration of 25(OH)D in the sample. Reference range of 25-hydroxycholecalciferol is shown in Table 1.

#### 2.2.2. Serum Calcium and Magnesium

Serum magnesium and total calcium levels were measured spectrophotometrically using ERBA-EM200 fully automated analyser and ERBA reagents:

 Reference range of serum magnesium: 1.7–2.5 mg/dL
 Reference range of serum calcium: 8.6–10.3 mg/dL

### 2.3. Statistical Methods

Results were tabulated in MS excel sheet and statistical analyses were carried out using the following methods:

(1) Descriptive statistics were used: for continuous variable range, mean (*x*) and standard deviation (sd) were calculated and for categorical variables proportion and percentage were obtained.
(2) Comparison between two sample proportions was done using the 2-sample *Z*-test and *P* values were calculated.
(3) To compare between variables of three or more groups, ANOVA test was applied.

## 3. Results

**Figure 1:**
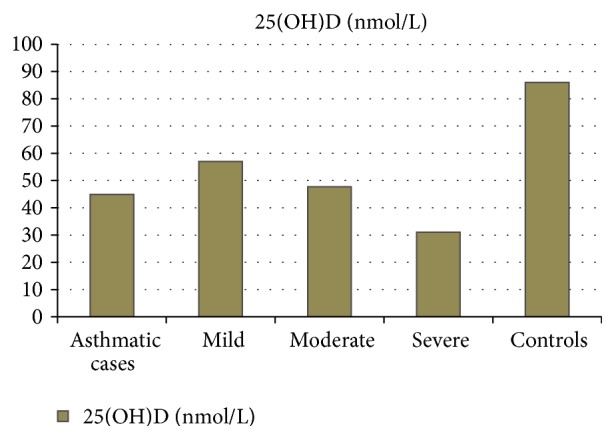
Vitamin D_3_ levels in different grades of asthma.

Table 2 shows demography of both the groups. Among the asthmatic patients (group I), 14 (23.3%) had mild persistent asthma, 27 (45%) had moderate persistent asthma, and 19 (31.7%) had severe persistent asthma. Severity of the disease was categorised according to GINA guidelines, 2016 [12]. The mean serum values of vitamin D and magnesium were significantly lower in patients of asthma, compared to healthy controls. Moreover results show stage-wise decline in serum levels of both vitamin D and magnesium with increase in severity of asthma. As shown in Table 6 and Figure 1, there was a negative correlation between serum vitamin D and magnesium levels with the grades of asthma severity. There was no significant difference in serum calcium levels between asthmatic patients (9.51 ± 0.54 mg/dL) and controls (9.62 ± 0.35 mg/dL).

As shown in Table 3, 63.3% asthmatic patients had vitamin D deficiency (<50 nmol/L) while 100% patients had insufficiency (<75 nmol/L). As far as serum magnesium levels are concerned, 58.8% asthmatic patients had hypomagnesemia (<1.7 mg/dL).

Table 4 shows serum magnesium levels in different grades of chronic stable asthma, mild persistent: 1.86 ± 0.07 mg/dL, moderate persistent: 1.70 ± 0.07 mg/dL, and severe persistent: 1.53 ± 0.09 mg/dL. This data clearly shows that serum levels of magnesium decrease with increase in severity of asthma.

As shown in Table 5, prevalence of hypomagnesemia and vitamin D deficiency increase with the increase in disease severity.

Table 6 shows that serum levels of magnesium and 25(OH)D decrease with the increase in disease severity as their levels are highest in mild form of asthma while lowest in severe persistent asthma (*P* < 0.001). Serum calcium levels were unaffected by decrease in other two analytes levels and so they do not affect severity of asthma (*P* = 0.52).

## 4. Discussion

Our study identifies the incidence of vitamin D insufficiency in terms of serum 25(OH)D <75 nmol/L (<30 ng/mL) and deficiency <50 nmol/L (<20 ng/mL) [13, 14]. This study shows that 63.3% of the patients had vitamin D deficiency and 100% had insufficiency, while, among the controls, 50% had insufficiency and 0% had deficiency. The mean level of 25(OH)D in group I asthma patients (44.9 ± 12 nmol/L) was significantly lower than group II controls (86 ± 13.3 nmol/L). Vitamin D status in asthma patients has been studied by many researchers. Bener et al. [6] reported the prevalence of vitamin D deficiency and hypomagnesemia in asthmatic children and concluded that vitamin D is strong predictor of asthma. Columbo et al. [15] showed 79% of the elderly asthmatic subjects had lower than normal serum vitamin D at baseline. These results confirm that vitamin D deficiency and insufficiency are extremely common in elderly patients with asthma and respiratory disease. Another study by Ginde et al. [16] identifies association of low vitamin D levels with higher frequency of respiratory infections and increased asthma severity. Brehm et al. [17] in their analysis found similar correlation. They found higher prevalence of allergic rhinitis with vitamin D deficiency. The reasons for widespread vitamin D deficiencies in various populations are not completely understood. However, high socioeconomic status and western lifestyle (indoor life and less exposure to sun) may contribute to vitamin D deficiency, which increases susceptibility to allergic diseases including bronchial asthma. In the current study patients had decreased serum levels of magnesium than controls. This could be explained by hypomagnesemia caused by repeated use of *β*_2_-agonist inhalation or nebulisation [18, 19] in turn causing vitamin D deficiency [20].

The current study shows relationship between vitamin D deficiency, hypomagnesemia, and severity of asthma. This was demonstrated in the levels of serum 25(OH)D in asthmatic patients, mild persistent: 57 ± 9.7 nmol/L, moderate persistent: 47.7 ± 6.2 nmol/L, and severe persistent: 31 ± 5.2 nmol/L, which is in agreement with Brehm et al. [17] and Sandhu and Casale [21], who reported that hypovitaminosis D increases the risk of severe exacerbation of asthma in children. Our study shows prevalence of hypomagnesemia in different grades of asthma, mild persistent: 1.86 ± 0.07 mg/dL (23.3%), moderate persistent: 1.70 ± 0.07 mg/dL (45%), and severe persistent: 1.53 ± 0.09 mg/dL (31.7%). This data shows that serum levels of magnesium and 25(OH)D decrease with the increase in disease severity. Their levels are highest in mild form of asthma while lowest in severe persistent asthma.

## 5. Conclusion

Vitamin D deficiency is prevalent in asthmatic patients. Moreover higher asthma severity, poor asthma control, and frequent exacerbations in asthmatic patients are associated with lower levels of vitamin D and magnesium. Serum 25(OH)D and magnesium levels may serve as markers of asthma severity. So levels of these analytes should be monitored in asthmatic patients and should be corrected if found low.

## Figures and Tables

**Table 1:** Ranges for 25-hydroxy vitamin D [13].

Interpretation	25(OH) D level
Normal	30–60 ng/mL
Deficiency	<20 ng/mL (<50 nmol/L)
Insufficiency	<30 ng/mL (<75 nmol/L)

**Table 2:** Distribution of patients and control groups.

	Group I	Group II
	Patients	Controls
	(*n* = 60)	(*n* = 60)
Gender		
Male	22 (36.7%)	30 (75%)
*Severity of asthma*		
Mild	4 (18.2%)	
Moderate	10 (45.5%)	
Severe	8 (36.4%)	
Females	38 (63.3%)	10 (25%)
*Severity of asthma*		
Mild	10 (26.3%)	
Moderate	17 (44.7%)	
Severe	11 (28.9%)	
Age (years)	40 ± 13	41 ± 10
BMI	23 ± 3.6	22 ± 1.4
History of asthma (years)	24 ± 7.2	
Familial history of asthma	12 (20%)	
Atopy	54 (90%)	None

**Table 3:** Serum 25(OH)D and magnesium levels in patients and controls.

		Patients	Controls	*Z* value	*P* value
Serum 25(OH)D	Deficient (<50 nmol/L)	38 (63.3%)	0 (0%)	6.4	<0.001
	Insufficient (<75 nmol/L)	60 (100%)	30 (50%)	6.1	
Magnesium	Normal	25 (41.2%)	57 (95%)	5.5	<0.001
	Hypomagnesemia (<1.7 mg/dL)	35 (58.8%)	3 (5%)	5.5	

**Table 4:** Serum magnesium levels in asthma.

Grades of asthma	Cases (*n* = 60)	S. magnesium level (mean ± sd)	ANOVA test *P* value
Mild	14 (23.3%)	1.86 ± 0.07	<0.001
Moderate	27 (45%)	1.70 ± 0.07	
Severe	19 (31.7%)	1.53 ± 0.09	

**Table 5:** Prevalence of hypomagnesemia and hypovitaminosis D in the different grades of asthma severity.

Grades of asthma	Hypomagnesemia (<1.7 mg/dL)	Hypovitaminosis D		Both (hypomagnesemia + vitamin D deficiency)
		Deficiency	Insufficiency	
Mild (*n* = 14)	5 (14.3%)	3 (21.4%)	14 (100%)	1 (7.1%)
Moderate (*n* = 27)	13 (48.1%)	16 (59.2%)	27 (100%)	10 (37%)
Severe (*n* = 19)	13 (68.4%)	19 (100%)	19 (100%)	13 (68.4%)
Total (60)	31 (51.7%)	38 (63.3%)	60 (100%)	24 (40%)

**Table 6:** Comparison of serum level of 25(OH)D, magnesium, and calcium in different grades of asthma.

	S. calcium (mg/dL)	S. magnesium (mg/dL)	25(OH)D (nmol/L)
Asthmatic cases			
Mild	9.44 ± 0.48	1.86 ± 0.07	57 ± 9.7
Moderate	9.46 ± 0.59	1.70 ± 0.07	47.7 ± 6.2
Severe	9.64 ± 0.51	1.53 ± 0.09	31 ± 5.2
Controls	9.62 ± 0.35	1.92 ± 0.1	86 ± 13.3
One-way ANOVA			
*F* value	0.81	32.8	122
*P* value	0.52	<0.001	<0.001
